# Medical cannabis use in the Australian community following introduction of legal access: the 2018–2019 Online Cross-Sectional Cannabis as Medicine Survey (CAMS-18)

**DOI:** 10.1186/s12954-020-00377-0

**Published:** 2020-06-08

**Authors:** Nicholas Lintzeris, Llewellyn Mills, Anastasia Suraev, Maria Bravo, Thomas Arkell, Jonathon C. Arnold, Melissa J. Benson, Iain S. McGregor

**Affiliations:** 1 Drug and Alcohol Services, South East Sydney Local Health District, Kogarah, NSW Australia; 2 Discipline of Addiction Medicine, Faculty Medicine and Health, University of Sydney, Sydney, NSW Australia; 3 The University of Sydney, Lambert Initiative for Cannabinoid Therapeutics, Sydney, NSW Australia; 4 Faculty of Science, School of Psychology, The University of Sydney, Sydney, NSW Australia; 5 Brain and Mind Centre, The University of Sydney, Sydney, NSW Australia; 6 Faculty of Medicine and Health, Central Clinical School, The University of Sydney, Sydney, NSW Australia; 7 Discipline of Pharmacology, Faculty of Medicine and Health, University of Sydney, Sydney, NSW Australia

**Keywords:** Medical cannabis, Medical marijuana, Epidemiology, Health policy

## Abstract

**Background:**

In 2016, the Australian federal government passed legislation enabling a range of cannabis-based products to be prescribed to patients by registered healthcare professionals. An online survey conducted immediately prior to these legislative changes found that the vast majority of respondents at the time were illicitly sourcing cannabis plant matter, smoking was the preferred route of administration and mental health, chronic pain, and sleep conditions were the most frequently cited reasons for medical cannabis use. This manuscript reports the results of a follow-up survey conducted in 2018–2019, the Cannabis As Medicine Survey (CAMS-18). The goal of this second questionnaire was to examine patterns of use and consumer perspectives regarding medical cannabis use in Australia, 2 years after the introduction of legal access pathways.

**Methods:**

Anonymous online cross-sectional survey with convenience sample, recruited mainly through online media between September 2018 and March 2019. Participants were adults (18 years or over) residing in Australia who reported using a cannabis product for self-identified therapeutic reasons during the preceding 12 months. The survey measured consumer characteristics, indications and patterns of medical cannabis use, routes and frequency of administration, perceived benefits and harms, experiences and preferred models of access to medical cannabis.

**Results:**

Data were available for 1388 respondents. The main categories of condition being treated with medical cannabis were pain (36.4%), mental health (32.8%), sleep (9.2%), neurological (5.2%) and cancer (3.8%). Respondents reported using medical cannabis on 15.8 (11.2) days in the past 28, by inhaled (71.4%) or oral (26.5%) routes and spending AUD$82.27 ($101.27) per week. There were high levels of self-reported effectiveness, but also high rates of side effects. There was uncertainty regarding the composition of illicit cannabinoid products and concerns regarding their possible contamination. Few respondents (2.7%) had accessed legally prescribed medical cannabis, with the main perceived barriers being cost, disinterest from the medical profession and stigma regarding cannabis use.

**Conclusions:**

Chronic pain, mental health and sleep remain the main clinical conditions for which consumers report using medical cannabis. Despite 2 years of legal availability, most consumers in Australia reported accessing illicit cannabis products, with uncertainty regarding the quality or composition of cannabis products.

## Introduction

The global trend towards the legalisation of cannabis for medical purposes reflects both the increased robustness of evidence supporting its efficacy [1] and increased interest amongst consumers in using cannabis-based therapeutics [2]. In this rapidly changing landscape, it is important for regulators and healthcare providers to understand community use of cannabis for medical purposes, and to determine how changes in medical cannabis legislation may impact patterns of use.

In November 2016, the Australian federal government passed legislation [3, 4] enabling a range of cannabis-based products to be prescribed as unregistered medicines using the Special Access and Authorised Prescriber Schemes [5, 6], and in December 2016 the Australian Therapeutic Goods Administration (TGA) published clinical guidance [7] regarding their use for a range of conditions. At the time of writing, more than 30,000 official approvals have been issued to allow patients to access to more than 100 different cannabis-based products including botanical material, oils and sprays provided by government-approved manufacturers and distributors [6, 8, 9]. Special Access Scheme (SAS-B) approvals cover a wide range of conditions but by far the largest category is chronic pain [10, 11]. Any medical practitioner can apply to the TGA under SAS-B for a product to treat an individual patient. Upon approval, cannabis-based products (developed under good manufacturing practice conditions) are dispensed to patients at pharmacies licensed to hold scheduled medicines. Given that the medical cannabis is an unregistered medicine, it is not subsidised by the government via the Pharmaceutical Benefits Scheme (PBS) or private health insurance schemes, and hence the patient must bear the cost of the medication which can be significant in some cases (typically $5–15 per day) [8, 12, 13].

Immediately prior to the legislative changes allowing access, our research group conducted an online consumer survey (‘Cannabis as Medicine Survey 2016’ or CAMS-16) of Australians who had indicated use of a cannabis-based product (either legally or illegally) for the management of a health condition in the preceding 12 months [14]. The vast majority of respondents at the time were illicitly sourcing cannabis plant matter, with smoking being the preferred route of administration. Only one respondent reported accessing medical cannabis on prescription. Mental health, chronic pain and sleep conditions were the most frequently cited reasons for medical cannabis use. Respondents generally reported high levels of clinical effectiveness, but also reported significant levels of, mostly minor, side effects.

Here, we report the results of a follow-up survey conducted approximately 2 years after the introduction of the 2016 legislative changes. The aim of the 2-year follow-up survey was to monitor changes in how Australians were accessing and using their medical cannabis following the 2016 legislative changes and the emergence, in the wake of these changes, of a more established medical cannabis environment, with increased community discussion and media attention and clearer federal guidelines to doctors around prescription and use of medical cannabis. The CAMS-18 survey, which recruited during the last quarter of 2018 and first quarter of 2019, involved many of the same questions as CAMS-16 to enable general comparisons to be made of consumer experiences over time, but also included refinement of various sections including extra questions regarding composition of cannabis products and perceptions of legal prescription cannabis models of access and care.

As with the CAMS-16 survey, the term ‘medical cannabis’ used in this paper refers to the term as understood by lay people—any licit or illicit cannabis-based product (including plant matter) used to treat or alleviate the symptoms of a self-identified health condition. This does not imply that the cannabis product was indicated or prescribed by a health professional.

## Methods

The study used a cross-sectional online survey design with a convenience sample of individuals self-reporting the use of cannabis for therapeutic reasons within the past 12 months. The study was approved by the University of Sydney Human Research Ethics Committee (2018/544). Survey questions examined the following areas:

- Medical conditions for which respondents reported using medical cannabis
- Current and lifetime patterns of medical and non-medical cannabis use, including source, route of administration, average frequency and cost
- Perceived benefits and harms associated with medical cannabis use, including side effects (symptom checklist); social and legal implications; and Patient Global Impression of Change (PGIC) [15], a 7-item patient-reported rating of symptom change
- The cannabinoid profile that respondents thought they were using (options of tetrahydrocannabinol (THC), cannabidiol (CBD) and THC:CBD combinations)
- Perspectives on accessing licit medical cannabis products—including the experiences of those who had accessed legally prescribed products, and reasons for not accessing prescribed products for respondents using only illicit products

The full CAMS-18 survey is included in online supplement 1.

Study data were collected and managed using Research Electronic Data Capture (REDCap), a secure web-based platform allowing respondents to directly enter responses online [16].

The CAMS-18 survey was freely accessible to any person who was supplied with the survey URL. The survey was ‘live’ online for 6 months (September 2018 to March 2019), and was promoted online using social media and consumer group webpages, and at consumer and professional forums. Eligibility criteria were (a) informed consent, (b) aged ≥ 18 years, (c) self-identified as a user of cannabis or a cannabinoid product for a medical purpose within the previous 12 months and (d) resident in Australia.

### Statistical analyses

Statistical analyses were performed in R version 3.4.1 [16] using the tidyverse [17], effsize [18], vector generalized linear and additive models (VGAM) [19] and rcompanion [20] packages. Only valid responses were analysed, with no imputation of missing data. As the number of valid responses varied across different items in the survey, categorical variable frequencies will be reported alongside the number of valid responses.

Differences between the CAMS-16 and CAMS-18 surveys were tested for several key variables, using independent samples *t* tests for continuous variables, chi-squared tests of independence for categorical variables and negative binomial regression for count variables. Where categorical variables had many levels, these levels were collapsed into fewer levels to aid interpretation of the chi-squared tests. Hedge’s *g* effects sizes were calculated for *t* tests (with rules of thumb: *g* < 0.2 = negligible, 0.2–0.5 = small, 0.5–0.8 = medium and *g* ≥ 0.8 = large) and Cramer’s *V* for chi-squared tests (rules of thumb: *V* < 0.1 = negligible, 0.1–0.3 = small, 0.3–0.5 = medium, *V* ≥ 0.5 large) [21, 22]. Due to the large sample sizes in both surveys, even very small differences between CAMS-16 and CAMS-18 were highly significant. Therefore, the results of statistical tests will be reported briefly in-text quoting effect size statistics only, with full details supplied in online supplement 2.

### Patient and public involvement

The CAMS-16 survey was extensively piloted with medical cannabis users through cooperation with cannabis user organisations across Australia. CAMS-16 item selection and survey design was thus heavily informed by consumer feedback. CAMS-18 was based on CAMS-16, with minor changes, and was piloted with a group of consumers reporting medical cannabis use for user-acceptance and ease of understanding of the questionnaire.

## Results

### Respondents

Of the 1804 respondents who commenced the survey, 184 did not meet eligibility criteria, and 192 did not give consent. Data were excluded for 70 respondents who provided no further information beyond demographics questions, three respondents who indicated that none of their cannabis use was for medical purposes and seven who provided implausible responses to numerous questions. Of the remaining 1388 respondents, 909 (65%) completed the entire survey.

Most respondents became aware of the survey via social media: 336/1387 (24.2%) through Facebook, and 838/1387 (59.5%) through other social media (e.g. Instagram, Twitter, Snapchat, Reddit, Whirlpool, Bluelight). Others were recruited through friends (4.7%, 65/1387), medical cannabis providers (1.8%, 25/1387), the website for the Lambert Initiative of Cannabinoid Therapeutics, a philanthropically funded research centre at the University of Sydney (1.7%, 23/1387), consumer groups (0.9%, 13/1387), traditional media (TV, radio, newspaper) (0.8%, 11/1387), doctors/healthcare providers (1.0%, 8/1387), cannabis access clinics (0.4%, 6/1387) and ‘other’ sources (4.5%, 62/1387). The proportion of respondents recruited through Facebook in CAMS-18 was much lower than in CAMS-16, and the proportion through other social media was much higher (*V* = 0.65).

### Respondent characteristics

Respondents’ characteristics are reported in Table 1. Respondents’ mean (± standard deviation) age was 43.4 ± 13.9 years and the majority were male (57.6%, 799/1387). Most respondents were employed (59.2%, 821/1387) and had attained either a trade/vocational certificate or a university degree (78.7%, 1092/1387). Compared to the CAMS-16 cohort, the CAMS-18 cohort were older and had proportionally greater numbers who (i) were female, (ii) were in a relationship and (iii) had a tertiary qualification; however, these demographic differences were small (*g* < 0.50 or *V* < 0.30) except for education level where there was a medium-sized effect (*V* = 0.30).

**Table 1:** Demographic characteristics of the CAMS-18 sample (*n* = 1387)

Characteristic	
Age, mean (SD)	43.4 (13.9)
Gender	
Female	560 (40.4%)
Male	799 (57.6%)
Other	28 (2.0%)
Relationship status	
Partnered (currently in relationship, including defacto and married)	861 (62.1%)
Single (not currently in a relationship, including separated, divorced, widowed)	526 (37.9%)
Indigenous status	
Aboriginal and/or Torres Strait Islander	56 (4.0%)
Not Aboriginal and/or Torres Strait Islander	1331 (96.0%)
Highest education level attained	
Primary school	14 (1.0%)
Secondary school	278 (20.0%)
Trade or vocational college	461 (33.2%)
University degree	631 (45.5%)
Other	3 (0.2%)
Employment status	
Full-time work	633 (45.6%)
Part-time work	188 (13.6%)
Home duties	78 (5.6%)
Student	77 (5.6%)
Unemployed	63 (4.5%)
Retired	128 (9.2%)

Missing values excluded from denominator when calculating percentages

### Cannabis use

Lifetime cannabis use history indicated that 19.1% (212/1109) had never used cannabis prior to using it for medical reasons, 35.7% (396/1109) reported previous non-medical cannabis use but had quit for 12 months or more prior to initiating medical cannabis use and 45.2% (501/1109) were using cannabis non-medically at the time they began using it medically. The proportion of respondents who had never used cannabis prior to using it for medical reasons was similar in both CAMS-16 and CAMS-18 (*V* = 0.07).

The mean estimated proportion of cannabis use for medical purposes (as a proportion of total use) was 83.2 ± 20.6% (Table 2). Respondents reported using medical cannabis on a median of 18 days in the past 28 days (IQR = 4, 28; mean = 15.8 ± 11.2).

**Table 2:** Patterns of cannabis use

Characteristic	*n*	
Age first tried cannabis for any reason, mean (SD)	1110	20.5 (11.6)
Age first regular cannabis use any reason, mean (SD)	1110	25.8 (16.3)
Age first regular cannabis use for medical reason, mean (SD)	1110	32.6 (17.5)
Never used cannabis regularly for any reason, *N* (%)	1110	134 (12.1%)
Number of days in previous 28 used cannabis for any reason	1110	
Mean (SD)		17.3 (10.9)
Median (IQR)		20 (5–28)
Number of days in previous 28 used cannabis for medical reasons	1110	
Mean (SD)		15.8 (11.2)
Median (IQR)		18 (4–28)
Estimated proportion of cannabis use for medical reasons, mean (SD)	1095	83.2% (20.6%)
Usual number of times using cannabis per day for any reason	1110	
Mean (SD)		3.3 (3.7)
Median (IQR)		2 (1–4)
Weekly cost of medical cannabis, mean (SD)	1101	$60.68 ($94.20)
Weekly cost of medical cannabis with respondents who did not pay excluded, mean (SD)	812	$82.27 ($101.27)

Median (IQR) reported for count variables only

*IQR* interquartile range

**Fig. 1:**
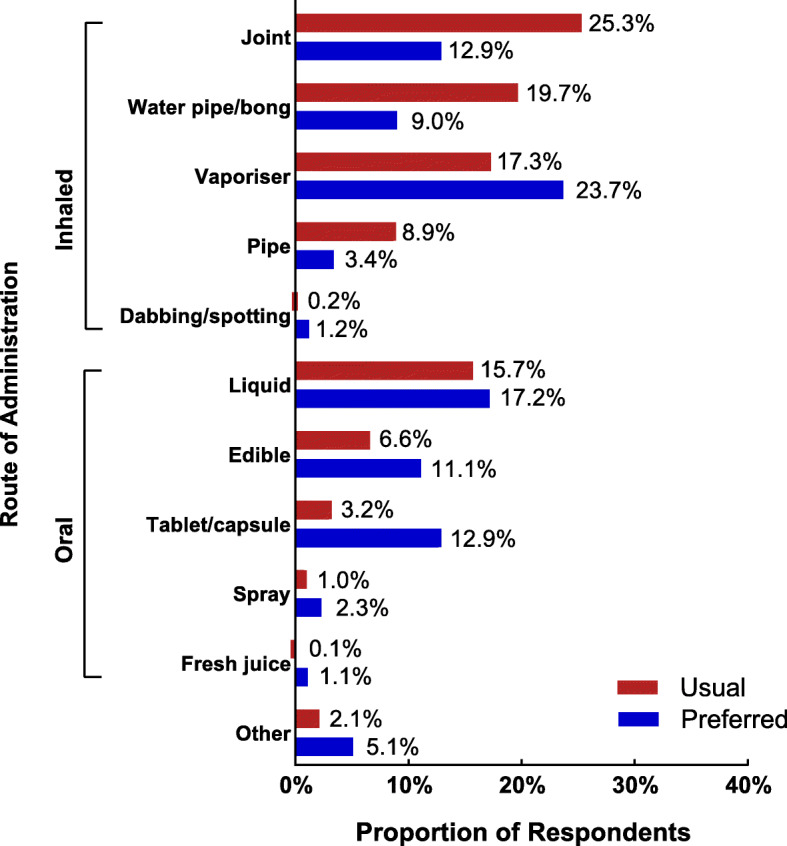
Usual and preferred methods of administering medical cannabis

Most respondents consumed their cannabis via an inhaled (71.4%; 788/1104) route (compared with oral [26.5%, 293/1104] or other [2.1%, 23/1104] routes); however, there was a stronger preference for oral or vaporised routes of administration over traditional smoked routes such as joints, pipes or bongs (Fig. 1). Compared to CAMS-16, a lower proportion of respondents in CAMS-18 indicated that they consumed and would prefer to consume their medical cannabis by inhalation, and a greater proportion indicated they consumed and would prefer to consume their medical cannabis orally; however, this effect was small (*V* = 0.15).

Compared to the CAMS-16 cohort, CAMS-18 respondents tended to (i) have started using cannabis later and used less cannabis for either medical or other reasons, and (ii) use a greater percentage of cannabis for medical purposes compared to non-medical purposes; however, these differences were all small to negligible (all *g* < 0.50).

### Composition of medical cannabis

Respondents reported they either did not know the composition of their cannabis (25.8%, 284/1103) or that it varied significantly between batches (23.9%, 264/1103). Further, 16.4% (181/1103) reported that their medical cannabis contained approximately equal levels of THC and CBD, 21.3% (235/1103) reported that it contained predominately THC (with either no, or small amounts of other cannabinoids), 12.2% (135/1103) reported that it contained predominately CBD and 0.4% (4/1103) reported ‘other’. Most (63.4%, 699/1105) were concerned about the possibility of contaminants (e.g. heavy metals, pesticides) in their cannabis.

### Conditions treated with medical cannabis

Respondents were asked to select from a structured list, up to five health conditions (‘Any condition’ column, Table 3), and the main condition that they had treated using medical cannabis. The categories most commonly endorsed for ‘Any condition’ were insomnia (41.5%, 573/1382), back pain (34.5%, 477/1382), anxiety (32.6%, 450/1382) and depression (27.9%, 386/1382). The most frequent main conditions were anxiety (12.6%, 168/1331), back pain (10.1%, 135/1331), depression (8.5%, 113/1331) and insomnia (7.1%, 94/1331).

**Table 3:** Conditions reported as reasons for using medical cannabis

Condition	Rank	Main condition^a^ (*n* = 1331)		Any condition^b^ (*n* = 1382)	
		Condition	*n* (%)^c^	Condition	*n* (%)^c^
Pain		Total	499 (37.5%)	Total	852 (61.6%)
	1	Back pain	135 (10.1%)	Back pain	477 (34.5%)
	2	Arthritis	79 (5.9%)	Arthritis	262 (19.0%)
	3	Nerve pain	75 (5.6%)	Headaches	215 (15.6%)
	4	Fibromyalgia	52 (3.9%)	Neck pain	202 (14.6%)
	5	All others	158 (12.0%)	All others	638 (46.1%)
Mental health		Total	437 (32.8%)	Total	621 (44.9%)
	1	Anxiety	168 (12.6%)	Anxiety	450 (32.6%)
	2	Depression	113 (8.5%)	Depression	386 (27.9%)
	3	PTSD	82 (6.2%)	PTSD	191 (13.8%)
	4	Bipolar affective disorder	15 (1.1%)	Addiction to other substances	67 (4.8%)
	5	All others	59 (4.5%)	All others	198 (14.3%)
Sleep		Total	123 (9.2%)	Total	679 (49.1%)
	1	Insomnia	94 (7.1%)	Insomnia	573 (41.5%)
	2	Circadian rhythm disorder	9 (0.7%)	Sleep movement disorder	140 (10.1%)
	3	Sleep movement disorder	9 (0.7%)	Circadian rhythm disorder	74 (5.4%)
	4	Parasomnia	1 (0.1%)	Sleep breathing disorder	55 (4.0%)
	5	All others	10 (0.8%)	All others	70 (5.1%)
Neurological		Total	69 (5.2%)	Total	147 (10.6%)
	1	Epilepsy	26 (2.0%)	Epilepsy	45 (3.3%)
	2	Autism	14 (1.1%)	Autism	27 (2.0%)
	3	Multiple Sclerosis	13 (1.0%)	Multiple Sclerosis	18 (1.3%)
	4	Dementia	1 (0.1%)	Dementia	6 (0.4%)
	5	All others	15 (1.2%)	All others	71 (5.2%)
Cancer		Total	50 (3.8%)	Total	106 (7.7%)
	1	Blood cancers	8 (0.6%)	Breast	19 (1.4%)
	2	Gastrointestinal cancers	6 (0.5%)	Skin	17 (1.2%)
	3	Brain	5 (0.4%)	Brain	15 (1.1%)
	4	Breast	5 (0.4%)	Reproductive	15 (1.1%)
	5	All others	26 (2.1%)	All others	57 (4.1%)
Gastrointestinal^d^		Total	40 (3.0%)	Total	175 (12.7%)
	1	Crohn’s disease	10 (0.8%)	Irritable Bowel Syndrome	101 (7.3%)
	2	Ulcerative colitis	10 (0.8%)	Ulcerative Collitis	25 (1.8%)
	3	Irritable Bowel syndrome	9 (0.7%)	Crohn’s Disease	19 (1.4%)
	4	All others	11 (0.8%)	All others	60 (4.3%)
Other		Total	113 (8.5%)	Total	165 (11.9%)
	1	Auto-immune condition	33 (2.5%)	Auto-immune condition	64 (4.6%)
	2	Gynaecological condition	25 (1.9%)	Skin condition	43 (3.1%)
	3	Infectious disease	9 (0.7%)	Respiratory conditions	41 (3.0%)
	4	Skin condition	9 (0.7%)	Gynaecological condition	31 (2.2%)
	5	All others	37 (2.9%)	All others	88 (6.5%)

^a^Respondents could only select one main condition that they treated with cannabis

^b^Respondents could select up to five conditions that they treated with cannabis

^c^Percentages displayed represent the proportion each specific category makes up of the entire available sample (i.e. *n*/1331 for main condition and *n*/1382 for any condition)

^d^There were only three specific conditions listed under the gastrointestinal group, and an ‘other’ category

The proportions of respondents who reported pain, mental health/substance use, sleep or other conditions as the main conditions they treated with MC were very similar across both CAMS-16 and CAMS-18 surveys (*V* = 0.06).

### Patient reports of symptoms being managed, effectiveness, side-effects and other adverse consequences

**Fig. 2:**
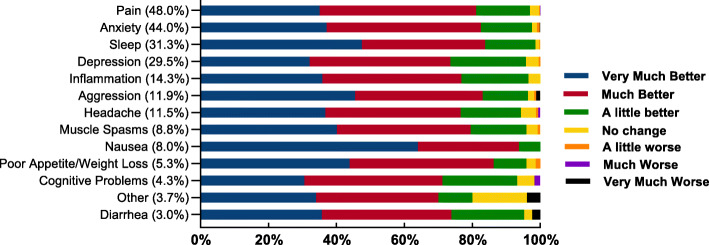
Most common symptoms treated with medical cannabis and change in those symptoms after treatment with medical cannabis

The symptoms that respondents reported being most often managed with medical cannabis mirrored the main conditions being treated (above section): pain (48.0%, 666/1388), anxiety (44.0%, 611/1388) and sleep problems (31.3%, 434/1388). The overwhelming majority of respondents reported symptom improvement following medical cannabis use (Fig. 2).

Side effects were commonly reported (Table 4), although relatively few reported these to be severe and/or intolerable. The most common mild and tolerable side effects were dry mouth (61.5%, 601/977), increased appetite (59.2%, 578/976), drowsiness (54.7%, 534/976) and eye irritation (30.2%, 294/974). The most common severe and/or intolerable side-effects were increased appetite (4.8%, 47/976), anxiety (2.4%, 23/974), dry mouth (2.4%, 23/977) and lack of energy or fatigue (2.1%, 20/973).

**Table 4:** Side-effect profile of medical cannabis use

Side-effect	Severity	
	Mild and tolerable	Severe and/or intolerable
Dry mouth (*n* = 977)	601 (61.5%)	23 (2.4%)
Increased appetite (*n* = 976)	578 (59.2%)	47 (4.8%)
Drowsy (*n* = 976)	534 (54.7%)	13 (1.3%)
Eye irritation (*n* = 974)	294 (30.2%)	8 (0.8%)
Lack of energy or fatigue (*n* = 973)	287 (29.5%)	20 (2.1%)
Anxiety (*n* = 974)	228 (23.4%)	23 (2.4%)
Memory impairment (*n* = 973)	227 (23.3%)	16 (1.6%)
Dehydration (*n* = 975)	220 (22.6%)	9 (0.9%)
Confusion (*n* = 975)	144 (14.8%)	9 (0.9%)
Respiratory complaints (*n* = 973)	141 (14.5%)	5 (0.5%)
Dizzy (*n* = 974)	137 (14.1%)	6 (0.6%)
Residual bad taste in mouth (*n* = 973)	135 (13.9%)	12 (1.2%)
Decreased Appetite (*n* = 975)	127 (13.0%)	8 (0.8%)
Paranoia (*n* = 973)	111 (11.4%)	9 (0.9%)
Racing heart or palpitations (*n* = 972)	106 (10.9%)	11 (1.1%)
Sweating (*n* = 974)	90 (9.2%)	10 (1.0%)
Depressed (*n* = 974)	87 (8.9%)	15 (1.5%)
Headaches (*n* = 973)	73 (7.5%)	11 (1.1%)
Sleep disturbance (*n* = 973)	72 (7.4%)	14 (1.4%)
Diarrhea (*n* = 974)	65 (6.7%)	7 (0.7%)
Constipation (*n* = 974)	52 (5.3%)	7 (0.7%)
Nasal Complaints (*n* = 973)	46 (4.7%)	8 (0.8%)
Gastro-intestinal irritation (*n* = 973)	44 (4.5%)	7 (0.7%)
Allergy (*n* = 974)	41 (4.2%)	5 (0.5%)
Panic Attacks (*n* = 973)	38 (3.9%)	8 (0.8%)
Shaking/tremor (*n* = 972)	37 (3.8%)	4 (0.4%)
Nausea/vomiting (*n* = 973)	36 (3.7%)	6 (0.6%)
Delusion (*n* = 974)	24 (2.5%)	4 (0.4%)
Hallucinations (*n* = 973)	22 (2.3%)	3 (0.3%)
Cannabis hyperemesis (*n* = 974)	12 (1.2%)	8 (0.8%)
Other (*n* = 970)	3 (0.3%)	11 (1.1%)

Almost half the respondents (47.6%, 448/942) indicated that the cost of medical cannabis placed a significant strain on their finances, 79.7% (751/942) worried about being arrested or other legal problems and 37.5% (353/942) were worried about employment issues. Further, 9.3% of respondents (88/942) reported that they had to undergo workplace drug testing.

### Accessing medical cannabis

When asked to list their main source of supply, 46.2% of respondents (482/1044) indicated that they obtained their medical cannabis from ‘recreational dealers’, 25.3% (264/1044) from friends or family, 11.6% (121/1044) by growing their own, 7.2% (75/1044) from illicit medicinal cannabis suppliers, 5.1% (53/1044) from online suppliers and 4.7% (49/1044) from ‘other’ sources. Only 2.4% of respondents (25/1044) indicated they had accessed licit medical cannabis prescribed by a doctor. These proportions were very similar to the proportions observed in the CAMS-16 survey (*V* = 0.14).

When asked why they had not accessed medical cannabis legally, 47.8% (433/906) of respondents indicated they did not know a medical practitioner willing to prescribe, 32.0% (290/906) were not aware they could access medical cannabis legally, 21.2% (192/906) indicated licit cannabis was too expensive, 18.4% (167/906) believed their medical practitioner was not interested or unwilling to prescribe cannabis, 12.7% (115/906) indicated they wanted their medical cannabis use to remain confidential from their healthcare providers, 9.5% (86/906) said they preferred illicit cannabis and 11.6% (105/906) gave other reasons.

One-quarter (26.2%, 289/1101) reported not paying for their cannabis, but indicated they were willing to pay a weekly mean (± SD) of AUD$38.33 ± 63.92 (median AUD$25, IQR: $10, $50) for prescribed products. Those respondents who did pay for cannabis estimated spending AUD$82.27 ± 101.27 per week (median $50, IQR: $20, $100; $12.24 less than respondents in CAMS-16, *g* = 0.13), and indicated that they were willing to pay AUD$68.67 ± 66.64 (median $50, IQR: $25, $100) for prescribed cannabis products.

### Seeking information about medical cannabis

When asked about their decision to try medical cannabis, most (51.5%, 523/1015) indicated that they discovered the benefits on their own (using cannabis and noticed symptoms improved), 10.5% (107/1015) reported internet-based media (e.g. Facebook, Reddit), 9.9% (100/1015) by a friend or family member, 6.5% (66/1015) by a medical cannabis advocacy group, 5.6% (57/1015) by a disease-specific consumer group, 5.0% (51/1015) by a healthcare provider and the remainder (10.9%, 111/1015) from other sources.

Although the initial interest in medical cannabis was generated by sources other than health professionals, most respondents (63.2%, 641/1015) had discussed their medical cannabis use with a healthcare provider, including their GP (83.6%, 536/641), medical specialist (54.3%, 348/641), psychologist (40.0%, 256/641), nurse (17.5%, 112/641), alternative medicine provider (17.2%, 110/641) and pharmacist (12.9%, 83/641).

### Accessing medically prescribed medical cannabis products

The 25 respondents who had accessed prescribed medical cannabis products had been accessing it for an average of 4.8 ± 3.8 months (median 3, IQR: 2, 6), prescribed by a medical specialist (64%; 16) or GP (36%, 9) for indications including fibromyalgia, multiple sclerosis, neuropathy, epilepsy, autism, Alzheimer’s, mesothelioma, post-traumatic stress disorder and back pain. Respondents estimated 18 ± 22.5 weeks (median 12, IQR: 4, 25) between their first cannabis-specific consultation with their doctor and receiving their first dose of medical cannabis. Although the numbers were too small to draw any firm conclusions, feedback from the 25 respondents who had accessed medical cannabis legally indicated generally positive ratings of their experience of product consistency (17 [68%] preferred licit supplies, 6 [24%] preferred illicit supplies, 2 [8%] no preference), ease of access (15 [60%] preferred licit to 7 [28%] illicit), cost (11 [44%] preferred licit to 8 [32%] illicit), effectiveness (11 [44%] preferred licit to 6 [24%] illicit), fewer side effects (13 [52%] preferred licit to 5 [20%] illicit) and legal status (20 [80%] preferred licit to 2 [8%] illicit).

### Attitudes to regulation of medical cannabis

Most respondents (78.3%, 721/921) indicated that people should be able to buy and use medical cannabis without approval by a medical practitioner, 92% (850) that medical cannabis should be part of routine healthcare in Australia, 70.7% (652) that the government should subsidise the cost of medical cannabis and 91.1% (839) that medical cannabis should meet safety standards (e.g. known strength, composition and contaminant-free). Most thought that the Australian regulatory framework for accessing medical cannabis did not work well (91.0%, 838/921), that the cost of licit medical cannabis was prohibitively expensive (62.6%, 577/921) and that the current model was difficult for patients to negotiate (87.3%, 804/921).

## Discussion

This survey provides a number of insights into medical cannabis use within the Australian community and updates our understanding of how consumer perspectives and behaviour have changed since the introduction of legal access pathways in November 2016. In many respects, little has changed in the 2 years since cannabis was legalised for medicinal purposes in Australia: users are still largely accessing illicit cannabis, self-medicating a similar range of health conditions (chronic pain, mental health and sleep problems), with similar perceived levels of effectiveness, side effects, social and legal issues reported. The findings that pain and mental health conditions remain the most common reasons for medical cannabis use and the generally high level of perceived efficacy is consistent with similar surveys of patients in jurisdictions with more established legal medical cannabis markets (e.g. Canada and various US states) [23–28].

The current survey recruited a slightly older and more educated cohort than CAMS-16. Respondents reported using cannabis on fewer days in the past 4 weeks and spent less per week on their medical cannabis than in CAMS-16; however, overall differences in patterns of use, conditions treated and attitudes of respondents between the two surveys are minor. CAMS-18 had slightly different recruitment strategies to CAMS-16, recruiting less from Facebook and more from Twitter, in part due to recent restrictions on advertising using the term ‘cannabis’ on Facebook. It is therefore difficult to know whether the small differences in key demographics and outcomes reflect a changing profile of Australian medical cannabis users or differences in the respondents sampled.

There is little in the current survey results to suggest that 2 years of legal medical cannabis access in Australia has transformed the ‘landscape’ of medical cannabis. The vast majority of respondents had not used the legal avenues available for prescription, with many respondents perceiving difficulties in finding medical practitioners willing to prescribe, and/or citing cost and stigma as barriers. Whilst few study respondents (*n* = 25) had accessed legal medical cannabis, those that had generally had more favourable perceptions regarding the legal form of the drug than those who had only ever used illicit forms. Interestingly, the small number of respondents who accessed legal medical cannabis tended to prefer it to illicit cannabis for its cost and ease of access. However, cost and ease of access were both endorsed as important *barriers* to accessing licit medical cannabis by respondents who had never obtained medical cannabis legally. This suggests that the barriers to licit use may involve a mistaken perception amongst illicit users (perhaps due to continuing public expressions of scepticism surrounding medical cannabis by some sectors of the medical profession and cannabis advocacy groups [29, 30]), rather than being the result of actual experiences following committed attempts to obtain access through legal channels. However, it should also be noted that as cannabis is currently an unregistered, unsubsidised medicine, patients must pay out of their own pocket for medications. Until medical cannabis products are licensed as medicines with the TGA, and subsidised under the Australian Pharmaceutical Benefits Scheme, it seems likely that the cost of unlicensed cannabis-based products will continue to force many people to source their medical cannabis illicitly [12], especially those on low incomes.

The predominant use of illicit sources of cannabis is consistent with the relatively limited number of official approvals under the TGA SAS-B at the time the survey was conducted. In the 6 months prior to September 2018, when the CAMS-18 survey opened, fewer than 1200 SAS-B approvals had been granted across Australia, and approximately 3000 approvals were granted during the study recruitment period (September 2018 to March 2019). Notably, however, in the 6-month period following the close of the CAMS-18 survey in March 2019, a further 13,000 approvals were issued [6] and total approvals as of January 2020 were around 30,000, involving more than 18,000 patients [6, 9]. Future CAMS surveys will attempt to explore this significant expansion in regulatory approvals and the impact upon medical cannabis consumers.

Our findings identify ongoing concerns regarding illicit supplies. As would be predicted with illicit products, there was scant knowledge of the composition of the products being used with regard to cannabinoid content (e.g. THC, CBD). This represents a fundamental issue given that the two cannabinoids have very distinct clinical indications and therapeutic effects. Specifically, CBD-only products having no intoxicating or euphorogenic properties, and when dosed appropriately have demonstrated efficacy in treating epilepsy, anxiety and psychosis [31, 32]. Even for those who thought they knew the composition of their cannabis products, it is worth noting that there is essentially no capacity for consumers to determine the strength or composition of illicit cannabis products in Australia, with no ability for laboratory testing of illicit cannabis products. In a previous study by our group, there was considerable discrepancy between perceived and actual cannabinoid profiles of illicit cannabis supplies used for children with epilepsy [33]. Similarly, almost two-thirds of respondents were worried about potential contaminants. Even in US states with long-established legal medical cannabis markets, recent studies suggest a disconcertingly high prevalence of inaccurately labelled cannabinoid products with significant over- and under-representation of THC and CBD content on products labels [34, 35]. Another health concern with legal medicinal cannabis products in these jurisdictions is the widespread use of ‘cannabis concentrates’ made using butane solvents and designed for ‘dabbing’ (vaporisation of a highly concentrated extract). This exposes the user to a highly potent THC levels (as high as 76%), and may also contain residual solvent [36]. Concerns around artisanal medical cannabis vaporisation products were also raised with recent reports of lung injury caused by vaporisation of contaminated illicit cannabis-based products in e-cigarettes [36, 37]. Clearly, these are not ideal conditions for any therapeutic intervention in a modern healthcare system.

Nonetheless, the move away from smoking (joints, bongs) to non-smoked cannabis-based products (vaporised cannabis, oral products) remains a positive trend in the current survey relative to CAMS-16, and is a trend that is evident in other countries [38–41]. In one recent survey of medical cannabis patients in Canada, most patients not only reported vaporisation as their primary route of administration but also indicated a preference for non-smoked routes over smoked routes [24]. Whilst, as noted above, vaporising does carry associated health concerns, the vaporisation of cannabis plant material is at least preferable to smoking, as the lower temperatures avoid production of the many toxic pyrolysis products that occurs when plant material is burned in joints or bongs.

Demand for medical cannabis products does not seem to be abating. The experience of consumers surveyed here suggests minimal uptake of licit and prescribed products during the first 2 years of official access in Australia, although there are indications that this is changing. The marked increase in SAS approvals since the close of this survey signals improved access to medical providers willing to engage in this area of medicine with more than 14,000 medical practitioners in Australia having now prescribed cannabis [9]. This has coincided with the emergence of a number of private clinics specialising in medicinal cannabis, which appears to have markedly simplified access to medicinal cannabis products for many patients. Recent surveys of Australian GPs [42] and specialists [43, 44] indicate that many medical practitioners feel relatively under-educated regarding this area of clinical practice. Over half of psychiatrists (54%) and GPs surveyed (57%) supported the availability of medical cannabis on prescription; however, a majority of GPs (52%) felt uncomfortable discussing medical cannabis with their patients, with over two-thirds of GPs reporting that they did not have good knowledge around medical cannabis. Lack of perceived knowledge on the topic is a common barrier for medical practitioners globally [42, 45–47], highlighting a need for improved training of medical practitioners around medical cannabis. Finally, whilst most respondents in our survey continued to express disappointment with the legal models of medical cannabis availability, those who had actually pursued the licit avenue reported quite positive experiences.

The study design has inherent limitations, as described in our previous CAMS-16 survey [14]. The reliance on self-report data is potentially associated with inaccurate information, such as incorrect diagnostic conditions, recall difficulties, or misinterpretation of effectiveness or adverse events. Furthermore, there is always likely to be a selection bias in any such survey towards recruiting people with favourable experiences of medical cannabis and cannabis legalisation generally. Whilst we were able to reduce the amount of missing data compared to the CAMS-16 survey, we acknowledge that valid responses to all questions were only available for 65% of respondents. Finally, the fact that CAMS-18 was recruited from a slightly different group of people to CAMS-16 makes it difficult to be sure whether the small differences in experiences relayed by the two cohorts were the result of changes in the medical cannabis landscape or simply differences in demographics.

## Conclusions

Our survey reflects the experiences of consumers during the first 2 years following major regulatory changes permitting medical cannabis access to patients in Australia. The early experiences of the small numbers of patients who had accessed legally prescribed products appear positive, although there remain many negative perceptions of access pathways amongst the vast majority of consumers who are not yet accessing these pathways. It remains to be seen how many of the individuals using illicit cannabis products for medical reasons legally will transfer to legally prescribed products over time. Until some form of medicinal-grade cannabinoid product is added to the list of medications subsidised by government (e.g. the Pharmaceutical Benefits Scheme) or private insurance schemes, cost seems likely to remain a significant barrier to widespread use of licit medical cannabis. Another potential concern is the many individuals who reported using medical cannabis for conditions for which there is little evidence [31, 48, 49] and no clinical guidance (e.g. management of anxiety). Given that many in the community are already using illicitly-obtained cannabis to treat their anxiety, depression and insomnia, there is an urgent need for more clinical trials to investigate the effectiveness of cannabis products for these conditions.

## Supplementary information

**Additional file 1.**

**Additional file 2.**

## Data Availability

The datasets used and/or analysed during the current study are available from the corresponding author on request.

## Abbreviations

AUD$: Australian Dollars
CAMS-16: Cannabis as Medicine Survey 2016
CAMS-18: Cannabis as Medicine Survey 2018
CBD: Cannabidiol
PGIC: Patient Global Impression of Change
PBS: Pharmaceutical Benefits Scheme
REDCap: Research Electronic Data Capture
SAS-B: Special Access Scheme
THC: Tetrahydrocannabinol
TGA: Therapeutic Goods Administration
VGAM: Vector generalized linear and additive models
