# Supplementary material for: Medical cannabis use in the Australian community following introduction of legal access: the 2018–2019 Online Cross-Sectional Cannabis as Medicine Survey (CAMS-18)
Source: Harm Reduct J. 2020 Jun 8;17:37. doi: 10.1186/s12954-020-00377-0 (PMC7278204; doi:10.1186/s12954-020-00377-0)
Supplement: Supplementary file 1 — Additional file 1. [file 12954_2020_377_MOESM1_ESM.pdf]

Cannabis as Medicine Survey: 2018

As researchers from the University of Sydney we are launching an Australia wide online survey of people who have used cannabis for MEDICAL (therapeutic) purposes within the last 12 months. A similar survey was conducted in 2016, and we aim to see how things have changed in that time.

The survey will be anonymous and confidential. You have been allocated a user ID code that in no way allows for your responses to be matched to personal and/or identifying data.

The survey asks about:

1. You: your age, gender, level of education and current living and work situations

2. Conditions you have used medical cannabis to treat/cure in the last 12 months

3. How you access and administer cannabis for medical purposes

4. The extent to which you experience benefits (e.g. symptom relief) and/or problems (e.g. side effects, social or legal problems) from your use of medical cannabis.

5. How you would like cannabis products to be available for medical reasons in the future

6. Your general health and recent use of other substances.

For further details and instructions please read the participant information statement attached here.

[Attachment: "ParticipantInformationStatement_CAMS18.pdf"]

Please indicate your eligibility for the study below:	◯ I am an Australian resident over the age of 18 who has used cannabis for MEDICAL reasons in the last 12 MONTHS. Click here to confirm.
Informed consent	◯ I have read the Participant Information Statement and understand the nature of my involvement in this study. I understand that I can withdraw from involvement at any time by exiting the server browser. In this case my data will not be saved or analysed. I also understand that I can save my responses and return to complete this study at a later time. To do so I must record the User ID code provided when I select the "save and return" option at the end of any page. Click here to agree.

SECTION 1

ABOUT YOU

Please provide some basic information about yourself.

1.1 Gender
- ◯ Male
- ◯ Female
- ◯ Other

1.1.a Please tell us which gender identity you most readily identify as.

_______________ (please specify)

1.2 Age

_______________ (Years old)

1.3 What is the post code of your current address?

____

1.4 Are you of Aboriginal or Torres Strait Islander origin?
- ◯ No
- ◯ Yes, Aboriginal
- ◯ Yes, Torres Strait Islander
- ◯ Yes, both Aboriginal and Torres Strait Islander

1.5 What is the highest level of education you have completed?
- ◯ Primary School
- ◯ Secondary/High school
- ◯ Trade of Vocational training (e.g. TAFE or college)
- ◯ University degree
- ◯ Other

How else would you describe your highest level of education?

_______________ (please describe)

1.6 Which option best describes your current employment status?
- ◯ Full time work
- ◯ Part time work
- ◯ Home duties
- ◯ Unemployed
- ◯ Retired
- ◯ Disability pension
- ◯ Student

1.7 Which of the following best describes the sector you have been employed in for the majority of your working life?
- ◯ Hospitality
- ◯ Retail
- ◯ Office
- ◯ Healthcare
- ◯ Construction
- ◯ Education
- ◯ Manufacturing
- ◯ Transport
- ◯ Mining
- ◯ Law enforcement
- ◯ Never employed
- ◯ Other

1.7.a How would you best describe the sector you have been employed in for the majority of your working life?

____

1.8 On how many days of the last 28 days did you engage in PAID work (do not include voluntary work) and/or study (school, TAFE, university or vocational training)?

____
(Please enter a value from 0-28 (Full time = 20 days))

1.9 What is your current relationship status?

◯ Single (not currently in a relationship)
◯ Partnered (currently in a relationship)

1.10 In the last 28 days, have you been the primary care giver for, or living with, any child/children under the age of 18?

◯ Yes
◯ No

1.11 Where did you first hear about this Survey?

◯ Facebook
◯ Other social media (e.g. Instagram, Twitter, Snapchat)
◯ Online forum (e.g. Reddit, Whirlpool, Bluelight)
◯ A friend
◯ Medical cannabis provider
◯ Consumer support group
◯ Lambert Initiative website
◯ Doctor/healthcare provider
◯ Cannabis Access Clinics
◯ Media (newspaper, radio, TV)
◯ Other
(select one option)

1.11.a From what other source did you first hear about this survey?

____
(please describe)

SECTION 2

REASONS FOR USING MEDICAL CANNABIS

The following questions ask about:

a) The medical condition(s) for which you use medical cannabis AND (b) the specific symptoms for which you use medical cannabis.

Please keep in mind that a medical condition refers to an injury, illness, or disease that affects your physical and/or mental health. In contrast, symptoms are what people experience (e.g. nausea, pain) and are often not specific to a medical condition.

EXAMPLE: Some people may have cancer as their main health CONDITION for using medical cannabis, and use cannabis for the relief of SYMPTOMS of pain, poor appetite, and anxiety.

2.1 Have you used medical cannabis to treat a condition from any of the following groups?

You may select more than one option

- ☐ Cancer
- ☐ Gastro-intestinal
- ☐ Mental health and/or substance use
- ☐ Neurological
- ☐ Pain
- ☐ Sleep
- ☐ Other

(select all that apply)

2.1.a Please indicate which of the following cancer conditions you have used medical cannabis to treat.

- ☐ Blood cancers (leukaemia, lymphoma, myeloma)
- ☐ Brain cancers (glioblastoma, neuroblastoma, mesothelioma)
- ☐ Breast cancer
- ☐ Gastrointestinal cancer (bowel, colon, stomach, pancreatic)
- ☐ Lung cancer
- ☐ Reproductive cancers (prostate, cervical, uterine, testicular, ovarian)
- ☐ Skin cancers (melanoma)
- ☐ Other forms of cancer

(select all that apply (limit of three))

What other type of cancer have you used medical cannabis to treat?

____

2.1.a Please indicate which of the following gastro-intestinal conditions you have used medical cannabis to treat.

- ☐ Crohn's disease
- ☐ Irritable bowel syndrome
- ☐ Ulcerative collitis
- ☐ Other gastro-intestinal condition

(select all that apply (limit of three))

What other type of gastro-intestinal conditions have you used medical cannabis to treat?

____

2.1.aPlease indicate which of the following mental health conditions you have used medical cannabis to treat.

- ☐ Addiction (Cannabis)
- ☐ Addiction (Other drugs: including alcohol, opioid, amphetamine)
- ☐ Anxiety Disorder (e.g. generalised anxiety, panic disorder, OCD)
- ☐ Attention Deficit disorder (ADHD)
- ☐ Depression
- ☐ Eating disorders (e.g. anorexia / bulimia/ obesity)
- ☐ Manic Disorder (Bipolar affective disorder)
- ☐ Post-Traumatic Stress Disorder
- ☐ Schizophrenia or other psychosis
- ☐ Other mental health condition

(select all that apply (limit of three))

2.1.bWhat other type of mental health condition do you use medical cannabis to treat?

____

2.1.aPlease indicate which of the following neurological conditions you use medical cannabis to treat

- ☐ Amyotrophic Lateral Sclerosis
- ☐ Autism
- ☐ Dementia (including Alzheimer's)
- ☐ Epilepsy/ Seizure disorder
- ☐ Glaucoma
- ☐ Huntington's disease
- ☐ Multiple Sclerosis
- ☐ Parkinson's Disease
- ☐ Tourette's Syndrome
- ☐ Other neurological condition

(select all that apply (limit of three))

2.1.aWhat other type of neurological conditions have you used medical cannabis to treat?

____
(please describe)

2.1.aPlease indicate which of the following pain conditions you have used medical cannabis to treat

- ☐ Arthritis (including rheumatoid or osteoarthritis
- ☐ Back pain
- ☐ Cancer-related pain
- ☐ Fibromyalgia
- ☐ Gynaecological pain
- ☐ Headaches (including migraines)
- ☐ Menstrual pain
- ☐ Neck pain
- ☐ Neuropathy (nerve pain)
- ☐ Spinal cord injury
- ☐ Other type of chronic, non-cancer pain not described above

(select all that apply (limit of three))

2.1.bWhat other type of pain disorder do you use medical cannabis to treat?

____
(please describe)

2.1.a Please indicate which of the following sleep conditions you have used medical cannabis to treat

- ☐ Circadian rhythm sleep disorder (e.g. shift work type)
- ☐ Insomnia (any type)
- ☐ Narcolepsy or other hypersomnia
- ☐ Parasomnias (e.g. sleep walking, nightmares)
- ☐ Sleep apnoea or other sleep-related breathing disorder
- ☐ Sleep-related movement disorder (e.g. restless leg syndrome)
- ☐ Other sleep disorder

(select all that apply (limit of three))

What other type of sleep disorder do you use medical cannabis to treat?

____
(please describe)

2.1.a Please indicate which of the following other conditions you use medical cannabis to treat

- ☐ Auto-Immune condition (e.g. SLE, Chronic Fatigue disorder)
- ☐ Cardiovascular condition (e.g. poor circulation, ischemic heart disease)
- ☐ Diabetes Mellitus
- ☐ Gynaecological condition (e.g. endometriosis, PMS)
- ☐ AIDS/HIV
- ☐ Infectious disease (e.g. viral hepatitis)
- ☐ Respiratory disease (e.g. asthma, cystic fibrosis)
- ☐ Skin condition (e.g. eczema, psoriasis, dermatitis)
- ☐ Other condition

(select all that apply (limit of five))

2.1.a What other conditions have you used medical cannabis to treat?

____
(please describe)

Of the conditions you have just listed, please indicate which is the MAIN (primary) health condition for which you have used cannabis for medical purposes:

2.2 MAIN health condition:

select one

◯ Addiction (Cannabis)
◯ Addiction (Other drugs: including alcohol, opioid, amphetamine)
◯ Amyotrophic Lateral Sclerosis
◯ Anxiety Disorder (e.g. generalised anxiety, panic disorder, OCD)
◯ Arthritis (including rheumatoid or osteoarthritis
◯ Attention Deficit disorder (ADHD)
◯ Autism
◯ Auto-Immune condition (e.g. SLE, Chronic Fatigue disorder)
◯ Back pain
◯ Blood cancers (leukaemia, lymphoma, myeloma)
◯ Brain cancers (glioblastoma, neuroblastoma)
◯ Breast cancer
◯ Cancer-related pain
◯ Cardiovascular condition (e.g. poor circulation, ischemic heart disease)
◯ Circadian rhythm sleep disorder (e.g. shift work type)
◯ Crohn's disease
◯ Dementia (including Alzheimer's)
◯ Depression
◯ Diabetes Mellitus
◯ Eating disorders (e.g. anorexia / bulimia/ obesity)
◯ Epilepsy/ Seizure disorder
◯ Fibromyalgia
◯ Gastrointestinal cancer (bowel, colon, stomach)
◯ Glaucoma
◯ Gynaecological condition (e.g. endometriosis, PMT)
◯ Headaches (including migraines)
◯ Huntington's disease
◯ Infectious disease (e.g. AIDS/HIV, viral hepatitis)
◯ Insomnia (any type)
◯ Irritable bowel syndrome
◯ Lung cancer
◯ Manic Disorder (Bipolar affective disorder)
◯ Menstrual pain
◯ Mesothelioma
◯ Multiple Sclerosis
◯ Narcolepsy or other hypersomnia
◯ Neck pain
◯ Neuropathy (nerve pain)
◯ Other forms of cancer
◯ Other gastro-intestinal condition
◯ Other mental health condition
◯ Other neurological condition
◯ Other sleep disorder
◯ Other type of chronic, non-cancer pain not described above
◯ Pancreatic cancer
◯ Parasomnias (e.g. sleep walking, nightmares)
◯ Post-Traumatic Stress Disorder
◯ Prostate cancer
◯ Reproductive cancers (cervical, uterine, testicular, ovarian)
◯ Respiratory disease (e.g. asthma, cystic fibrosis)
◯ Schizophrenia or other psychosis
◯ Skin cancers (melanoma)
◯ Skin condition (e.g. eczema, psoriasis, dermatitis)
◯ Sleep apnoea or other sleep-related breathing disorder
◯ Sleep-related movement disorder (e.g. restless leg syndrome)
◯ Spinal cord injury
◯ Tourette's Syndrome
◯ Ulcerative collitis
◯ Other condition not mentioned above
(select one option)

	What other health condition is your MAIN reason for using medical cannabis?	__________________________________<br>(please describe one condition)
2.3	Please check the option that best describes how your MAIN health condition is now compared with how it was before using medical cannabis?	◯ Very much better<br>◯ Much better<br>◯ A little better<br>◯ No change<br>◯ A little worse<br>◯ Much worse<br>◯ Very much worse<br>(select one option)

CANNABIS FOR SYMPTOM MANAGEMENT

2.4.1 Please indicate which of the following SYMPTOMS you have used medical cannabis to manage in the past 12 months (Choose up to three symptoms)

- ☐ Anxiety
- ☐ Agitation
- ☐ Breathing (respiratory) problems
- ☐ Cognitive problems (e.g. problems with memory, concentration)
- ☐ Depression
- ☐ Diarrhoea/constipation
- ☐ Drug withdrawal
- ☐ Headache
- ☐ Inflammation
- ☐ Muscle spasms
- ☐ Nausea vomiting
- ☐ Occular pressure
- ☐ Pain
- ☐ Poor appetite/weight loss
- ☐ Psychosis
- ☐ Seizures
- ☐ Skin
- ☐ Sleep
- ☐ Other symptom

(select all that apply)

2.4.2 Please rate how your anxiety has changed since using medical cannabis:

- ◯ Very much better
- ◯ Much better
- ◯ A little better
- ◯ No change
- ◯ A little worse
- ◯ Much worse
- ◯ Very much worse

(select one option)

2.4.2 Please rate how your agitation has changed since using medical cannabis:

- ◯ Very much better
- ◯ Much better
- ◯ A little better
- ◯ No change
- ◯ A little worse
- ◯ Much worse
- ◯ Very much worse

(select one option)

2.4.2 Please rate how your respiratory symptoms you have changed since using medical cannabis:

- ◯ Very much better
- ◯ Much better
- ◯ A little better
- ◯ No change
- ◯ A little worse
- ◯ Much worse
- ◯ Very much worse

(select one option)

2.4.2 Please rate how your cognitive problems have changed since using medical cannabis

- ◯ Very much better
- ◯ Much better
- ◯ A little better
- ◯ No change
- ◯ A little worse
- ◯ Much worse
- ◯ Very much worse

(select one option)

2.4.2Please rate how your depression has changed since using medical cannabis:

◯ Very much better
◯ Much better
◯ A little better
◯ No change
◯ A little worse
◯ Much worse
◯ Very much worse
(select one option)

2.4.2Please rate how your diarrhoea/constipation has changed since using medical cannabis:

◯ Very much better
◯ Much better
◯ A little better
◯ No change
◯ A little worse
◯ Much worse
◯ Very much worse
(select one option)

2.4.2Please rate how your symptoms of drug withdrawal have changed since using medical cannabis:

◯ Very much better
◯ Much better
◯ A little better
◯ No change
◯ A little worse
◯ Much worse
◯ Very much worse
(select one option)

2.4.2Please rate how your headaches have changed since using medical cannabis:

◯ Very much better
◯ Much better
◯ A little better
◯ No change
◯ A little worse
◯ Much worse
◯ Very much worse
(select one option)

2.4.2Please rate how your Inflammation has changed since using medical cannabis:

◯ Very much better
◯ Much better
◯ A little better
◯ No change
◯ A little worse
◯ Much worse
◯ Very much worse
(select one option)

2.4.2Please rate how your muscle spasms have changed since using medical cannabis:

◯ Very much better
◯ Much better
◯ A little better
◯ No change
◯ A little worse
◯ Much worse
◯ Very much worse
(select one option)

2.4.2Please rate how your nausea/vomiting has changed using medical cannabis:

◯ Very much better
◯ Much better
◯ A little better
◯ No change
◯ A little worse
◯ Much worse
◯ Very much worse
(select one option)

2.4.2Please rate how your symptoms of occular pressure have changed since using medical cannabis:

◯ Very much better
◯ Much better
◯ A little better
◯ No change
◯ A little worse
◯ Much worse
◯ Very much worse
(select one option)

2.4.2Please rate how your pain has changed since using medical cannabis:

◯ Very much better
◯ Much better
◯ A little better
◯ No change
◯ A little worse
◯ Much worse
◯ Very much worse
(select one option)

2.4.2Please rate how your appetite/weight has changed since starting medical cannabis:

◯ Very much better
◯ Much better
◯ A little better
◯ No change
◯ A little worse
◯ Much worse
◯ Very much worse
(select one option)

2.4.2Please rate how your Psychosis has changed since using medical cannabis:

◯ Very much better
◯ Much better
◯ A little better
◯ No change
◯ A little worse
◯ Much worse
◯ Very much worse
(select one option)

2.4.2Please rate how your seizures have changed since using medical cannabis:

◯ Very much better
◯ Much better
◯ A little better
◯ No change
◯ A little worse
◯ Much worse
◯ Very much worse
(select one option)

2.4.2 Please rate how your skin complaints have changed since using medical cannabis:

◯ Very much better
◯ Much better
◯ A little better
◯ No change
◯ A little worse
◯ Much worse
◯ Very much worse
(select one option)

2.4.2 Please rate how your sleep problems have changed since using medical cannabis:

◯ Very much better
◯ Much better
◯ A little better
◯ No change
◯ A little worse
◯ Much worse
◯ Very much worse
(select one option)

2.4.2 Please describe any other symptoms you have used medical cannabis to treat/cure

____
(Please indicate NO MORE than three other symptoms)

2.4.3 For your MAIN other symptom (choose one), how has this symptom changed since starting medical cannabis?

◯ Very much better
◯ Much better
◯ A little better
◯ No change
◯ A little worse
◯ Much worse
◯ Very much worse
(select one option)

SECTION 3: PATTERNS OF CANNABIS USE

3.1 How old were you when you first tried cannabis for ANY reason?

__________________ (years old (in numbers))

3.2 How old were you when you first tried cannabis for MEDICAL purposes?

__________________ (years old (in numbers))

3.2.calc

____

The age you entered as the age you first used cannabis for MEDICAL reasons is lower than the age you first tried cannabis for ANY reason. This does not make sense. Please think carefully and adjust your answers accordingly.

3.3 How old were you when you first started using cannabis REGULARLY for ANY reason? (at least once per week for a period of three months or longer).

If you have never used cannabis REGULARLY please enter '0'

__________________ (years old (in numbers). If you have never used cannabis regularly please enter '0')

3.3.calc

____

The age you entered as the age you first used cannabis REGULARLY is lower than the age you entered as the age you FIRST TRIED cannabis. This does not make sense. Please think carefully and adjust your answers accordingly.

3.4 How old were you when you first started using cannabis REGULARLY for MEDICAL purposes? (at least once per week for a period of three months or longer).

If you have never used cannabis REGULARLY for MEDICAL reasons please enter '0'

__________________ (years old (in numbers). If you have never used cannabis regularly for medical reasons please enter '0')

____

____

The age you entered as the age you first used cannabis REGULARLY for MEDICAL purposes is lower than the age you entered as the age you FIRST used cannabis for MEDICAL purposes. This does not make sense. Please think carefully and adjust your answers accordingly.

3.4.calc

____

The age you entered as the age you first used cannabis REGULARLY for MEDICAL reasons is lower than the age you entered as the age you first used cannabis REGULARLY for ANY reason (medical reasons are included in any reason). This does not make sense. Please think carefully and adjust your answers to these questions accordingly.

3.4.calc

____

In the previous question you entered '0', indicating that you had NEVER used cannabis for ANY reason REGULARLY, yet you have given a value greater than 0 for the age you began using cannabis REGULARLY for MEDICAL reasons. This does not make sense. Please think carefully and adjust your answers to these questions accordingly.

3.5 Which of the following best describes your use of cannabis before you used it for medical reasons?

- ◯ I had not used cannabis before using it for medical reasons
- ◯ I had used cannabis for non-medical reasons but had quit for a year or more before taking up cannabis use for medical reasons.
- ◯ I was using cannabis for non-medical purposes at the time I started using it medically.

(select one option)

3.5.a Please estimate the time, in months, between your last non-medical use of cannabis and starting medical cannabis

____
(number of months (e.g. if one year and two months enter '14'))

3.6 In the last 28 days, please estimate how many days you used cannabis for ANY reason:

____
(enter (0-28))

3.7 On an average day that you use cannabis for ANY reason, on how many occasions do you usually use cannabis (how many times a day)?

____
((max 20))

3.8 In the last 28 days, please estimate how many days you used cannabis for MEDICAL reasons:

____
(enter (0-28))

3.8.a

____

The value you entered for number of days using cannabis for MEDICAL reasons is greater than your entry for the number of days using cannabis for ANY reason (i.e. including medical). This does not make sense (days' use MEDICAL can only ever be equal to or less than days' use for ANY reason). Please adjust your answers to these questions accordingly.

3.9 On an average day that you use cannabis for MEDICAL reasons, on how many occasions do you usually use cannabis (how many times a day)?

____
((max 20))

3.9.a

____

The value you entered for number of times per day you use cannabis for MEDICAL reasons is greater than the value you entered for number of times per day for ANY reason. This does not make sense (times used per day for MEDICAL reasons must always be equal to or less than times per day for ANY reason). Please adjust your answers to these questions accordingly.

3.10 On the days that you use cannabis, please indicate the percentage of use you consider to be MEDICAL, as a proportion of your total use for ANY reason:

0% 50% 100%

(Place a mark on the scale above)

3.11 In the last twelve months have you had a break of one month or more from using cannabis for medical reasons?

- ◯ Yes
- ◯ No

3.11.a Why did you stop using medical cannabis?

- ☐ Unable to find supply
- ☐ Could not afford it
- ☐ Did not like the side effects (including the 'high')
- ☐ It did not work
- ☐ I no longer suffered from my health complaint
- ☐ I wanted a break from cannabis use
- ☐ Other

(select all that apply)

3.11.b Please tell us the other reason you stopped using medical cannabis

____
(please describe)

3.12 Please estimate the cost of your MEDICAL cannabis on an average WEEKLY basis in Australian dollars.

If you do not purchase your medical cannabis, or if you donate money towards your medical cannabis please enter zero (0).

____
(amount per week in dollars)

3.13 What type of medical cannabis do you MAINLY use?

- ◯ THC only
- ◯ Mainly THC and small amount of other cannabinoids
- ◯ Approximately equal amounts of THC and CBD
- ◯ Mainly CBD and small amounts of other cannabinoids (e.g. THC)
- ◯ CBD only
- ◯ Unsure/Do not know
- ◯ It varies between batches
- ◯ Other

(select one option)

3.13.a What other type of cannabinoid is in the medical cannabis you use?

____
(please describe)

3.14 Do you feel that the quality or composition of your medical cannabis is consistent over time?

- ◯ Yes
- ◯ No
- ◯ Unsure/Don't know

(select one option)

3.15 Are you concerned about the possibility of contaminants in your cannabis? (e.g. heavy metals, toxins, pesticides, bacteria/fungi/mould)

- ◯ Not at all
- ◯ A little
- ◯ Not sure
- ◯ Quite a bit
- ◯ A great deal

(select one option)

3.16 What is the MAIN way you consume cannabis for MEDICAL purposes?

◯ Oral (tablet or capsule)
◯ Oral edibles (cake, cookie etc.)
◯ Oral liquid (tincture, etc.)
◯ Oral spray (mouth or oromucosal)
◯ Fresh juice
◯ Nasal application (through nose)
◯ Rolled into a joint
◯ Pipe (plastic, metal, glass)
◯ Water pipe/ Bong
◯ Vaporiser
◯ Suppository
◯ Dabbing or spotting
◯ Topical (cream or patch applied to the skin)
◯ Other
(select one option)

3.16.a What other way have you consumed cannabis?

____
(please describe one other way of consuming cannabis)

3.17 When using your usual dose of medical cannabis how long does it take for you to feel ANY effect?

____
(please enter a number of MINUTES (1 hr = 60min))

3.18 When using your usual dose of medical cannabis how long does it take to achieve its MAXIMUM effect (e.g. relief from symptoms)?

____
(please enter a number of MINUTES (1 hr = 60min))

3.18calc

____

The time you entered as the time it takes for you to feel the MAXIMUM effect of cannabis is lower than the time you entered as the time it takes you to feel ANY effect. This does not make sense. Please adjust your answers accordingly.

3.19 When using your usual dose of medical cannabis how long would you estimate its effects last for (i.e. how long before you no longer feel any effects)?

____
(please enter a number of MINUTES (1 hr = 60min))

____

The time you entered as the time it takes until you NO LONGER FEEL ANY EFFECTS is less than the time you entered as the time it takes for you to feel ANY effect. This does not make sense. Please adjust your answers to these questions accordingly.

ACCESS TO MEDICAL CANNABIS

3.20 In the last 12 MONTHS, which of the following ways have you used to access your medical cannabis supply?

You may select more than one option

- ☐ I grow my own
- ☐ From friends or family
- ☐ From a recreational dealer
- ☐ From a medicinal cannabis supplier (not prescribed by a medical practitioner)
- ☐ From a 'club' or 'co-operative' outlet
- ☐ From an online supplier
- ☐ From an overseas supplier
- ☐ Prescribed by a medical practitioner and dispensed from a pharmacy
- ☐ Other

(select all that apply)

3.20.a Which other ways have you used to access medical cannabis?

____
(please describe)

3.21 In the last 12 MONTHS, what is the MAIN way you accessed your medical cannabis?

Select one option only

- ◯ I grow my own
- ◯ From friends or family
- ◯ From a recreational dealer
- ◯ From a medicinal cannabis supplier (not prescribed by a medical practitioner)
- ◯ From a 'club' or 'co-operative' outlet
- ◯ From an online supplier
- ◯ From an overseas supplier
- ◯ Prescribed by a medical practitioner and dispensed from a pharmacy
- ◯ Other

(select all that apply)

3.21.a What is the MAIN way you have used to access medical cannabis?

____
(Please provide a brief description)

3.22 In the last 28 days have you ever been unable to access medical cannabis to treat your health concerns?

- ◯ Yes
- ◯ No

3.23 Do you worry about accessing your preferred source of cannabis?

- ◯ Very much so
- ◯ Somewhat
- ◯ No opinion
- ◯ Not especially
- ◯ Not at all

(select one option)

SECTION 4: MEDICAL CANNABIS, Health Care and Social Issues

4.1 Which of the following MOST informed your decision to use medical cannabis for your health condition?

- ◯ Discovered benefits on my own (i.e. was using cannabis and noticed my symptoms improved)
- ◯ Internet-based media (e.g. Facebook, Reddit, Instagram)
- ◯ Conventional media (e.g. television, radio, newspaper, magazine)
- ◯ Conventional healthcare provider (e.g. GP, medical specialist, pharmacist, nurse, counsellor)
- ◯ Alternative healthcare practitioner (e.g. herbalist, naturopath)
- ◯ Friend or family member
- ◯ Consumer group focused on a particular condition (e.g. epilepsy, chronic pain, mental health)
- ◯ Medical cannabis advocacy group (e.g. United in Compassion, Hemp embassy)
- ◯ Other

(select one option)

4.1.a What other source most informed your decision to use medical cannabis for your condition?

____

4.2 Have you discussed your medical cannabis use with any of your healthcare providers?

- ◯ Yes - with most of my healthcare providers
- ◯ Yes- but only with a small number of my healthcare providers
- ◯ No - not with any of my healthcare providers

(select one option)

4.2.a Please indicate which healthcare providers you have discussed your medical cannabis use with

- ☐ General Practitioner (GP)
- ☐ Specialist medical practitioner (e.g. pain specialist, neurologist, oncologist, psychiatrist)
- ☐ Counsellor/psychologist
- ☐ Nurse
- ☐ Pharmacist
- ☐ Alternative therapist (e.g. acupuncturist, herbalist)
- ☐ Other

(select all that apply)

4.2.b Please tell us what other kind of healthcare provider you have discussed your medical cannabis use with.

____

(please describe)

4.2.b Which of the following best describes your healthcare provider's general attitude to your use of medical cannabis?

- ◯ All are supportive of my medical cannabis use
- ◯ Most (but not all) are supportive of my medical cannabis use
- ◯ Uncertain
- ◯ Most (but not all) do not support my medical cannabis use
- ◯ All do not support my medical cannabis use

(select one option)

4.3 Do you feel comfortable disclosing the details of your medical cannabis use with close friends/family?

- ◯ Yes - to most of my friends/family
- ◯ Yes - but only to some close friends/family members
- ◯ No

(select one option)

4.3.a Which of the following best describes your family and friends' general attitude to your use of medical cannabis

- ◯ All are supportive of my medical cannabis use
- ◯ Most (but not all) are supportive of my medical cannabis use
- ◯ Uncertain
- ◯ Most (but not all) do not support my medical cannabis use
- ◯ All do not support my medical cannabis use

(select one option)

4.4 Have you used the following medications to treat any of the health conditions for which you use medical cannabis?

- ☐ Opioids (e.g. morphine, oxycodone, codeine, tramadol, fentanyl, hydromorphone, buprenorphine, methadone)
- ☐ Benzodiazepines or other hypnotics (e.g. diazepam, oxazepam, clonazepam, alprazolam, temazepam, nitrazepam, lorazepam, zopiclone, zolpidem)
- ☐ Antidepressants (e.g. fluoxetine, escitalopram, venlafaxine, amitryptilline, mirtazapine)
- ☐ Antipsychotic medication (e.g. quetiapine, chlorpromazine, olanzipine, amisulpiride)
- ☐ Anticonvulsants (e.g. carbamazepine, valproate, phenytoin, lamotrigine, topiramate, levetiracetam, clonazepam, lacosamide)
- ☐ Gabapentinoids (e.g. Gabapentin, Pregabalin)
- ☐ Non-Steroidal Anti-Inflammatories (e.g. ibuprofen, paracetamol)
- ☐ Other

(select all that apply)

4.4.a You indicated you have used Opioids to treat health conditions that you now use medical cannabis to treat. Which of the following best describes how your use of Opioids has changed since using medical cannabis?

Since using medical cannabis my use (either frequency and/or dose) of Opioids has:

- ◯ Markedly decreased (by 50% or more)
- ◯ Moderately decreased (by 20-50%)
- ◯ More or less stayed the same (no change greater than 20% each way)
- ◯ Moderately increased (by 20-50%)
- ◯ Markedly increased (by 50%)

(select one option)

4.4.a You indicated you have used Benzodiazepenes to treat health conditions that you now use medical cannabis to treat. Which of the following best describes how your use of Benzodiazepenes has changed since using medical cannabis?

Since using medical cannabis my use (either frequency and/or dose) of Benzodiazepenes has:

- ☐ Markedly decreased (by 50% or more)
- ☐ Moderately decreased (by 20-50%)
- ☐ More or less stayed the same (no change greater than 20% each way)
- ☐ Moderately increased (by 20-50%)
- ☐ Markedly increased (by 50%)

(select one option)

4.4.a You indicated you have used Antidepressants to treat health conditions that you now use medical cannabis to treat. Which of the following best describes how your use of Antidepressants has changed since using medical cannabis?

Since using medical cannabis my use (either frequency and/or dose) of Antidepressants has:

- ☐ Markedly decreased (by 50% or more)
- ☐ Moderately decreased (by 20-50%)
- ☐ More or less stayed the same (no change greater than 20% each way)
- ☐ Moderately increased (by 20-50%)
- ☐ Markedly increased (by 50%)

(select one option)

4.4.a You indicated you have used Antipsychotic medications to treat health conditions that you now use medical cannabis to treat. Which of the following best describes how your use of Antipsychotic medications has changed since using medical cannabis?

Since using medical cannabis my use (either frequency and/or dose) of Antipsychotics has:

- ☐ Markedly decreased (by 50% or more)
- ☐ Moderately decreased (by 20-50%)
- ☐ More or less stayed the same (no change greater than 20% each way)
- ☐ Moderately increased (by 20-50%)
- ☐ Markedly increased (by 50%)

(select one option)

4.4.a You indicated you have used Anticonvulsants to treat health conditions that you now use medical cannabis to treat. Which of the following best describes how your use of Anticonvulsants medications has changed since using medical cannabis?

Since using medical cannabis my use (either frequency and/or dose) of Anticonvulsants has:

- ☐ Markedly decreased (by 50% or more)
- ☐ Moderately decreased (by 20-50%)
- ☐ More or less stayed the same (no change greater than 20% each way)
- ☐ Moderately increased (by 20-50%)
- ☐ Markedly increased (by 50%)

(select one option)

4.4.a You indicated you have used Gabapentinoids to treat health conditions that you now use medical cannabis to treat. Which of the following best describes how your use of Gabapentinoid medications has changed since using medical cannabis?

Since using medical cannabis my use (either frequency and/or dose) of Gabapentinoids has:

- ☐ Markedly decreased (by 50% or more)
- ☐ Moderately decreased (by 20-50%)
- ☐ More or less stayed the same (no change greater than 20% each way)
- ☐ Moderately increased (by 20-50%)
- ☐ Markedly increased (by 50%)

(select one option)

4.4.a You indicated you have used Non-Steroidal Anti-Inflammatories (e.g. ibuprofen, paracetamol) to treat health conditions that you now use medical cannabis to treat. Which of the following best describes how your use of Non-Steroidal Anti-Inflammatories medications has changed since using medical cannabis?

Since using medical cannabis my use (either frequency and/or dose) of non-steroidal anti-inflammatories (e.g. ibuprofen, paracetamol) has:

- ☐ Markedly decreased (by 50% or more)
- ☐ Moderately decreased (by 20-50%)
- ☐ More or less stayed the same (no change greater than 20% each way)
- ☐ Moderately increased (by 20-50%)
- ☐ Markedly increased (by 50%)

(select one option)

4.4.a What other medication have you used to treat the symptoms you now treat with medical cannabis?

____
(please list one other medication)

4.4.a How has your use of this other medication has changed since using medical cannabis?

Since using medical cannabis my use of this medication has:

- ☐ Markedly decreased (by 50% or more)
- ☐ Moderately decreased (by 20-50%)
- ☐ More or less stayed the same (no change greater than 20% each way)
- ☐ Moderately increased (by 20-50%)
- ☐ Markedly increased (by 50%)

(select one option)

4.5 On how many of the last 28 days have you consumed Tobacco?

____
((0-28))

4.5.a Please estimate how many cigarettes (of tobacco) you currently consume per day

____
(number of cigarettes per day)

4.5.b Since starting medical cannabis my use of tobacco has:

◯ Markedly decreased (by 50% or more)
◯ Moderately decreased (by 20-50%)
◯ More or less stayed the same (no change greater than 20% each way)
◯ Moderately increased (by 20-50%)
◯ Markedly increased (by 50% or more)
(select one option)

4.6 On how many of the last 28 days did you consume alcohol

____
((0-28))

4.6.a Please estimate how many standard drinks you consumed per day (on days when you drank).

Guide:
285 ml of beer, 425 of light beer, 100ml of wine, 60ml of fortified wine and 30 ml of spirits, all = 1 standard drink.

A full-strength can or stubby of beer = 1.5 standard drinks.

____
(In standard drinks.)

4.6.b Since starting use of medical cannabis, my use of alcohol has:

◯ Markedly decreased (by 50% or more)
◯ Moderately decreased (by 20-50%)
◯ More or less stayed the same (no change greater than 20% either way)
◯ Moderately increased (by 20-50%)
◯ Markedly increased (by 50% or more
(select one option)

4.7 SIDE EFFECTS

Indicate below whether you have experienced any of the following side effects in the last 12 months when using medical cannabis, and, if so, how serious these side effects were.

Note: 'Mild and Tolerable' = symptoms that caused only mild impairment of function and do not require specific treatment (e.g. other medications or stopping medical cannabis); 'Severe and/or intolerable' = symptoms that caused impairment of function, and/or resulted in either other treatment (e.g. other medications) or resulted in stopping medical cannabis use.

	No	Mild and tolerable	Severe and/or intolerable
Allergies	○	○	○
Anxiety	○	○	○
Cannabis hyperemesis	○	○	○
Confusion	○	○	○
Constipation	○	○	○
Decreased appetite	○	○	○
Dehydration	○	○	○
Delusions	○	○	○
Depressed mood	○	○	○
Diarrhoea	○	○	○
Dizziness	○	○	○
Drowsiness or sedation	○	○	○
Dry mouth	○	○	○
Eye irritation (red, sore or itchy eyes)	○	○	○
Gastro-intestinal irritation	○	○	○
Hallucinations	○	○	○
Headaches	○	○	○
Increased appetite	○	○	○
Lack of energy or fatigue	○	○	○
Memory impairment	○	○	○
Nasopharyngeal complaints (e.g. sinusitis, pharyngitis)	○	○	○
Nausea and/or vomiting	○	○	○
Panic attack	○	○	○
Paranoia	○	○	○
Racing heart or palpitations	○	○	○
Residual bad taste in mouth	○	○	○
Respiratory complaints (e.g. bronchitis, cough)	○	○	○

Shaking/tremor/difficulty controlling movements	○	○	○
Sleep disturbance	○	○	○
Sweating	○	○	○
Other	○	○	○

Please indicate what other type of side effect you have experienced.

____
(please describe)

4.8 CONSEQUENCES OF MEDICAL CANNABIS USE

Please indicate below whether you have experienced any of the following IN THE PAST 12 MONTHS

	Yes	No
I often take cannabis in larger amounts or over a longer period of time than I intended to	○	○
I have a persistent desire, or make unsuccessful attempts to cut down or control my cannabis use	○	○
I spend a great deal of time in activities necessary to obtain cannabis, use cannabis, or recover from its effects	○	○
I have cravings, or a strong desire or urge to use cannabis	○	○
My cannabis use results in failure to fulfil my major role obligations at work, school, or home	○	○
I continue to use cannabis despite having persistent or recurrent social or interpersonal problems related to cannabis use (such as criminal charges, ultimatums of abandonment form spouses/partners/friends, and poor productivity	○	○
I have given up or reduced important social, occupational, or recreational activities because of cannabis use	○	○
I recurrently used cannabis in situations in which it is physically hazardous (e.g. driving motor vehicle, operating machinery)	○	○
I continued to use cannabis even though it causes problems with emotions, mental, or physical health (e.g. cough)	○	○

I needed to use cannabis more than before to get the desired effects or the same amount of cannabis has less effects than before	○	○

4.9 EXPERIENCES WHEN STOPPING CANNABIS USE

Within a week of stopping cannabis use that has been heavy or prolonged (i.e. usually daily or almost daily use over a period of at least a few months) I develop symptoms of:

	Yes	No
Irritability, anger or aggression	○	○
Nervousness or anxiety	○	○
Sleep difficulty (e.g., insomnia, disturbing dreams)	○	○
Decreased appetite or weight loss	○	○
Restlessness	○	○
Depressed mood	○	○
Significant distress from at least one of the following: abdominal pain, shakiness/tremors, sweating, fever, chills or headache	○	○
I take cannabis (or a closely related substance) to relieve or avoid withdrawal symptoms	○	○

4.10 FINANCIAL/LEGAL IMPLICATIONS

4.10 Does the cost of accessing your medical cannabis place a significant strain on your finances?
◯ Yes
◯ No

4.11 Does your medical cannabis use make you worry about being arrested or other legal problems?
◯ Yes
◯ No

4.12 Does your medical cannabis use make you worry about the security of your employment?
◯ Yes
◯ No

4.13 Do you have to do workplace drug testing
◯ Yes
◯ No

4.11 DRIVING

4.14 Have you driven a motor vehicle in the past 12 months?
- ◯ Yes
- ◯ No

4.15 After using medical cannabis, how long do you typically wait before driving?
- ◯ I drive within one hour of using cannabis
- ◯ I wait 1-3 hours after using cannabis
- ◯ I wait 4-6 hours after using cannabis before driving
- ◯ I wait 7-12 hours after using cannabis before driving
- ◯ I wait 13-24 hours after using cannabis before driving
- ◯ I wait at least 24 hours after using cannabis before driving

4.16 In the last 12 months, have you undergone road side testing by the police when having used medical cannabis?
- ◯ Yes
- ◯ No

4.17 Does the presence of roadside drug testing deter you from driving after you have consumed medical cannabis?
- ◯ Yes
- ◯ No

4.18 Have you been convicted of driving under the influence of cannabis IN THE LAST 12 MONTHS?
- ◯ Yes
- ◯ No

4.19skip Q4.19 skipped

4.19 Have you EVER been convicted of driving under the influence of cannabis?
- ◯ Yes
- ◯ No

4.19 Have you EVER been convicted of driving under the influence of cannabis?
- ◯ Yes
- ◯ No

4.20 Thinking about how you feel after consuming medical cannabis, please indicate to what extent you agree or disagree with the following statements:

4.20.a I am slower to react to sudden situations
- ◯ Strongly Disagree
- ◯ Disagree
- ◯ Neither agree nor disagree
- ◯ Agree
- ◯ Strongly agree

4.20.b I find it harder to remain focused
- ◯ Strongly Disagree
- ◯ Disagree
- ◯ Neither agree nor disagree
- ◯ Agree
- ◯ Strongly agree

4.20.c I tend to drive more carefully
- ◯ Strongly Disagree
- ◯ Disagree
- ◯ Neither agree nor disagree
- ◯ Agree
- ◯ Strongly agree

4.20.d I find it harder to stick to the speed limit
◯ Strongly Disagree
◯ Disagree
◯ Neither agree nor disagree
◯ Agree
◯ Strongly agree

4.20.e I tend to leave a larger gap between me and the car ahead
◯ Strongly Disagree
◯ Disagree
◯ Neither agree nor disagree
◯ Agree
◯ Strongly agree

4.20.f I find myself taking more risks
◯ Strongly Disagree
◯ Disagree
◯ Neither agree nor disagree
◯ Agree
◯ Strongly agree

4.20.g I find myself drifting out of my lane
◯ Strongly Disagree
◯ Disagree
◯ Neither agree nor disagree
◯ Agree
◯ Strongly agree

4.20.h I feel more in control of the vehicle
◯ Strongly Disagree
◯ Disagree
◯ Neither agree nor disagree
◯ Agree
◯ Strongly agree

4.20.i I am confident that I can accurately assess my driving ability
◯ Strongly Disagree
◯ Disagree
◯ Neither agree nor disagree
◯ Agree
◯ Strongly agree

4.21 Do you think medical cannabis impairs your driving ability?
◯ Yes
◯ No
(select one option)

SECTION 5: ACCESS AND ATTITUDES TO MEDICAL CANNABIS

ACCESS

5.1 Have you accessed legal medical cannabis prescribed by a doctor in Australia?

- ◯ Yes
- ◯ No

5.1.a Please describe the main reasons for you not accessing medical cannabis from a medical practitioner

- ☐ I did not know I could legally access medical cannabis from a doctor
- ☐ I do not know a medical practitioner who is willing to prescribe medical cannabis
- ☐ My medical practitioner is not interested or is unwilling to prescribe
- ☐ Medical cannabis supplies are too expensive for me to afford
- ☐ I prefer using illicit cannabis
- ☐ I prefer to keep my medical cannabis use confidential from healthcare providers
- ☐ Other reason

5.1.a.1 What is the other reason you have not accessed cannabis through a medical provider?

____

5.1.a For how long have you been accessing medically prescribed cannabis?

Please answer in number of MONTHS

____
(in months (please use numbers))

5.1.b What is the main indication (medical condition) for which you are prescribed medical cannabis?

(select one option)

- ◯ Addiction (Cannabis)
- ◯ Addiction (Other drugs: including alcohol, opioid, amphetamine)
- ◯ Amyotrophic Lateral Sclerosis
- ◯ Anxiety Disorder (e.g. generalised anxiety, panic disorder, OCD)
- ◯ Arthritis (including rheumatoid or osteoarthritis
- ◯ Attention Deficit disorder (ADHD)
- ◯ Autism
- ◯ Auto-Immune condition (e.g. SLE, Chronic Fatigue disorder)
- ◯ Back pain
- ◯ Blood cancers (leukaemia, lymphoma, myeloma)
- ◯ Brain cancers (glioblastoma, neuroblastoma)
- ◯ Breast cancer
- ◯ Cancer-related pain
- ◯ Cardiovascular condition (e.g. poor circulation, ischemic heart disease)
- ◯ Circadian rhythm sleep disorder (e.g. shift work type)
- ◯ Crohn's disease
- ◯ Dementia (including Alzheimer's)
- ◯ Depression
- ◯ Diabetes Mellitus
- ◯ Eating disorders (e.g. anorexia / bulimia/ obesity)
- ◯ Epilepsy/ Seizure disorder
- ◯ Fibromyalgia
- ◯ Gastrointestinal cancer (bowel, colon, stomach)
- ◯ Glaucoma
- ◯ Gynaecological condition (e.g. endometriosis, PMT)
- ◯ Headaches (including migraines)
- ◯ Huntington's disease
- ◯ Infectious disease (e.g. AIDS/HIV, viral hepatitis)
- ◯ Insomnia (any type)
- ◯ Irritable bowel syndrome
- ◯ Lung cancer
- ◯ Manic Disorder (Bipolar affective disorder)
- ◯ Menstrual pain
- ◯ Mesothelioma
- ◯ Multiple Sclerosis
- ◯ Narcolepsy or other hypersomnia
- ◯ Neck pain
- ◯ Neuropathy (nerve pain)
- ◯ Other forms of cancer
- ◯ Other gastro-intestinal condition
- ◯ Other mental health condition
- ◯ Other neurological condition
- ◯ Other sleep disorder
- ◯ Other type of chronic, non-cancer pain not described above
- ◯ Pancreatic cancer
- ◯ Parasomnias (e.g. sleep walking, nightmares)
- ◯ Post-Traumatic Stress Disorder
- ◯ Prostate cancer
- ◯ Reproductive cancers (cervical, uterine, testicular, ovarian)
- ◯ Respiratory disease (e.g. asthma, cystic fibrosis)
- ◯ Schizophrenia or other psychosis
- ◯ Skin cancers (melanoma)
- ◯ Skin condition (e.g. eczema, psoriasis, dermatitis)
- ◯ Sleep apnoea or other sleep-related breathing disorder
- ◯ Sleep-related movement disorder (e.g. restless leg syndrome)
- ◯ Spinal cord injury
- ◯ Tourette's Syndrome
- ◯ Ulcerative collitis
- ◯ Other condition not mentioned above

5.1.b1 What other condition not mentioned in the list above is the MAIN condition for which you are prescribed medical cannabis?

____

5.1.c Who is the main prescriber of your medical cannabis?

◯ GP
◯ Medical specialist
(select one option)

5.1.d Please estimate how long it took you to get your first dose of medical cannabis from the time you approached your medical cannabis doctor.

Please answer in number of WEEKS

If it took more than six months please enter '25'

____
(time in weeks (please use numbers))

5.1.e Please indicate your preference for illicit versus prescribed medical cannabis for each of the following

5.1.e1 Consistency of dose from batch to batch

◯ Strongly prefer illicit
◯ Prefer illicit
◯ No preference
◯ Prefer prescribed
◯ Strongly prefer prescribed
(select one option)

5.1.e2 Ease of access (getting supplies)

◯ Strongly prefer illicit
◯ Prefer illicit
◯ No preference
◯ Prefer prescribed
◯ Strongly prefer prescribed
(select one option)

5.1.e3 Cost

◯ Strongly prefer illicit
◯ Prefer illicit
◯ No preference
◯ Prefer prescribed
◯ Strongly prefer prescribed
(select one option)

5.1.e4 Effectiveness (how well it treats my health condition)

◯ Strongly prefer illicit
◯ Prefer illicit
◯ No preference
◯ Prefer prescribed
◯ Strongly prefer prescribed
(select one option)

5.1.e5 Side effects

◯ Strongly prefer illicit
◯ Prefer illicit
◯ No preference
◯ Prefer prescribed
◯ Strongly prefer prescribed
(select one option)

5.1.e6 Security re: legal status

◯ Strongly prefer illicit
◯ Prefer illicit
◯ No preference
◯ Prefer prescribed
◯ Strongly prefer prescribed
(select one option)

ATTITUDES

5.2 What should be the legal status of cannabis in Australia?

- ◯ Cannabis should be legal for ALL purposes (medical and other)
- ◯ Cannabis should be legal for MEDICAL purposes ONLY
- ◯ Cannabis should be ILLEGAL for ALL reasons
- ◯ Uncertain

5.3 If you could legally access medical cannabis, which form of administration would you prefer to use?

- ◯ Oral (tablet or capsule)
- ◯ Oral edibles (cake, cookie etc.)
- ◯ Oral liquid concentrate (tincture, etc.)
- ◯ Oral liquid as a fresh juice
- ◯ Oral mouth spray
- ◯ Nasal application (through nose)
- ◯ Smoked as a 'joint' (cigarette)
- ◯ Smoked using a 'dry' pipe (plastic, metal, glass)
- ◯ Smoked using a water pipe or 'Bong'
- ◯ Smoked by 'dabbing' or 'spotting'
- ◯ Inhaled using a vaporiser
- ◯ Suppository
- ◯ Topical (cream or patch applied to the skin)
- ◯ Other

(select one option)

5.3.a What other form of administration would be your preferred choice for using medical cannabis?

____

5.4 How would you prefer to legally access medical cannabis products?

- ◯ Grow your own
- ◯ Trade/buy from a friend
- ◯ From a special licensed cannabis dispensary
- ◯ From a pharmacy like any other medication
- ◯ No preference, so long as a regular supply is accessible
- ◯ Don't know

5.5 Please provide an estimation of how much you would be willing to pay per WEEK for legal access to medical cannabis?

____
(In Australian dollars)

5.6 ATTITUDES TO REGULATION OF MEDICAL CANNABIS

Please indicate the degree to which you agree/ disagree with each of the following statements regarding medical cannabis:

	Strongly disagree	Disagree	Neither agree or disagree	Agree	Strongly agree
A person should be able to buy and use medical cannabis without approval by a medical practitioner	○	○	○	○	○
A person should only be able to legally use medical cannabis if a medical professional prescribes it for them	○	○	○	○	○
Medical cannabis should be considered as part of routine health care in Australia	○	○	○	○	○
Medical cannabis should meet safety standards (e.g. be of known strength, composition and be free of contaminants)	○	○	○	○	○
The cost of medical cannabis should be subsidised by the government	○	○	○	○	○
The current regulatory framework in Australia for accessing medical cannabis works well	○	○	○	○	○
The cost of accessing legal medical cannabis in Australia is prohibitively expensive	○	○	○	○	○
The current model for accessing medical cannabis in Australia is 'straightforward' / 'easy to negotiate' for patients	○	○	○	○	○

SECTION 6:

6.1 GENERAL HEALTH and QUALITY OF LIFE

The following three questions assess your psychological health, physical health, and overall quality of life over the last 28 days.

Please answer on a scale from 0 to 10, with 0 being very poor and 10 being very good.

	0 Poor	1	2	3	4	5	6	7	8	9	10 Good
How do you rate your overall PSYCHOLOGICAL health? (consider mood, emotions, and feelings)	○	○	○	○	○	○	○	○	○	○	○
How do you rate your overall PHYSICAL health? (consider physical symptoms and/or illness)	○	○	○	○	○	○	○	○	○	○	○
How do you rate your overall QUALITY OF LIFE? (e.g. ability to enjoy life, getting on well with family and friends, satisfied with living conditions)	○	○	○	○	○	○	○	○	○	○	○

6.2 EUROHIS-QOl 8 Item

The following 8-item questionnaire also assesses your quality of life

	Very Poor	Poor	Neither	Good	Very good
How would you rate your quality of life?	◯	◯	◯	◯	◯

	Very dissatisfied	Dissatisfied	Neither satisfied nor dissatisfied	Satisfied	Very satisfied
How satisfied are you with your health?	◯	◯	◯	◯	◯

	Not at all	A little	Moderately	Mostly	Completely
Do you have enough energy for everyday life?	◯	◯	◯	◯	◯
Have you enough money to meet your needs	◯	◯	◯	◯	◯

	Very dissatisfied	Dissatisfied	Neither satisfied nor dissatisfied	Satisfied	Very satisfied
How satisfied are you with your ability to perform your daily living activities	◯	◯	◯	◯	◯
How satisfied are you with yourself?	◯	◯	◯	◯	◯
How satisfied are you with your personal relationships	◯	◯	◯	◯	◯
How satisfied are you with the conditions of your living space	◯	◯	◯	◯	◯

Is there anything we have not asked that you would like to add in regards to your experience of medical cannabis? (Please do not disclose identifying information such as names)	__________________________________
Please feel free to attach any further comments/feedback on this study	
