# Supplementary material for: Medical cannabis use in the Australian community following introduction of legal access: the 2018–2019 Online Cross-Sectional Cannabis as Medicine Survey (CAMS-18)
Source: Harm Reduct J. 2020 Jun 8;17:37. doi: 10.1186/s12954-020-00377-0 (PMC7278204; doi:10.1186/s12954-020-00377-0)
Supplement: Supplementary file 2 — Additional file 2. [file 12954_2020_377_MOESM2_ESM.docx]

Medical cannabis use in the Australian community following introduction of legal access:

The 2018-2019 Online Cross-Sectional Cannabis as Medicine Survey (CAMS-18)

**Supplementary Materials 2**

**Table 1:** Statistical tests of differences between CAMS16 and CAMS18 continuous or count variables

| **Variable** | **Test** | **Scale** | **Summary**  **Form** | ***N*** | | **Summary** | | **Stat Type** | **Stat**  **Value** | ***p*** | **Group**  **Diff** | **95% CI** | | **Effect Size** |
| --- | --- | --- | --- | --- | --- | --- | --- | --- | --- | --- | --- | --- | --- | --- |
|  |  |  |  | **C16** | **C18** | **C16** | **C18** |  |  |  |  | **Low** | **High** |  |
| Age | Welch  unpaired  *t*-test | yrs-old | M (SD) | 1744 | 1388 | 37.9 (13.4) | 43.4 (13.9) | *t* | -11.1 | <0.001 | -5.5 | -6.4 | -4.5 | *g*^a^=-0.40 |
| Age first use ANY reason | Welch  unpaired  *t*-test | yrs-old | M (SD) | 1183 | 1110 | 18.6 (8.9) | 20.5 (11.6) | *t* | -4.5 | <0.001 | -1.9 | -2.8 | -1.1 | *g*=-0.19 |
| Age first use MEDICAL reason | Welch  unpaired  *t*-test | yrs-old | M (SD) | 1444 | 1111 | 27.4 (13.0) | 35.4 (14.3) | *t* | -14.5 | <0.001 | -7.9 | -9.0 | -6.9 | *g*=-0.58 |
| Age first REGULAR use ANY reason | Welch  unpaired  *t*-test | yrs-old | M (SD) | 1159 | 1110 | 23.7 (11.0) | 25.8 (16.3) | *t* | -3.5 | <0.001 | -2.1 | -3.2 | -0.9 | *g*=-0.15 |
| Age first REGULAR use MEDICAL reason | Welch  unpaired  *t*-test | yrs-old | M (SD) | 1400 | 1107 | 29.2 (12.8) | 32.6 (17.5) | *t* | -5.4 | <0.001 | -3.4 | -4.6 | -2.2 | *g*=-0.23 |
| Percentage of cannabis used for medical reasons | Welch  unpaired  *t*-test | % | M (SD) | 1421 | 1095 | 81.2 (19.8) | 83.2 (20.6) | *t* | -2.5 | 0.013 | -2.0 | -3.6 | -0.4 | *g*=-0.10 |
| Days used cannabis in previous 28 days (any reason) | Welch  unpaired  *t*-test | Days used in last 28 | M (SD) | 1429 | 1104 | 20.9 (9.4) | 17.3 (10.9) | *t* | 8.8 | <0.001 | 3.6 | 2.8 | 4.4 | *g*=0.36 |
| Days used cannabis in previous 28 days (medical reasons) | Welch  unpaired  *t*-test | Days used in last 28 | M (SD) | 1443 | 1105 | 19.8 (10.0) | 15.8 (11.2) | *t* | 9.4 | <0.001 | 4.0 | 3.2 | 4.8 | *g*=0.38 |
| On day when cannabis used for ANY reason number of times per day cannabis is used | Zero-truncated  negative binomial regression | Number of times per day | Median (IQR) | 1134 | 1095 | 3 (2,6) | 2 (1,4) | irr | 0.55^a^ | <0.001 | - | 0.49 | 0.62 | - |
| Weekly cost of MC (excluding respondents who did not pay for cannabis) | Welch  unpaired  *t*-test | $AUD | M (SD) | 909 | 812 | $94.51 ($86.56) | $82.27 ($101.27) | *t* | 2.7 | 0.007 | $12.24 | $3.28 | $21.20 | *g*=0.13 |

*g = Hedge’s g effect size estimate. Rules of thumb for effect size: g< 0.2 = negligible, g < 0.5 = small, g < 0.8 = medium, g 0.8 ≥ large (Cohen, 1992). Irr = incidence rate ratio.*

**Table 2:** Statistical tests of differences between CAMS16 and CAMS18 Categorical Variables

| **Variable** | **Test** | **Summary**  **Form** | ***N*** | | **Group** | **Summary** | | **Statistic** | ***p*** | **Effect Size** |
| --- | --- | --- | --- | --- | --- | --- | --- | --- | --- | --- |
|  |  |  | **C16** | **C18** |  | **C16** | **C18** |  |  |  |
| Recruitment source | Chi-squared | *n* (%) | 1783 | 1387 | Facebook  Other Social Media  A friend  Other | 1437 (81%)  67 (4%)  51 (3%)  228 (12%) | 336 (24%)  838 (60%)  65 (5%)  148 (11%) | $\chi^{2}$(3)=1330.5 | <0.001 | V=0.65 |
| Sex | Chi-squared | *n* (%) | 1748 | 1387 | Female  Non-Female | 545 (31%)  1203 (69%) | 560 (40%)  827 (60%) | $\chi^{2}$(1)=28.3 | <0.001 | V=0.10 |
| In a relationship | Chi-squared | *n* (%) | 1725 | 1387 | In a relationship  Not in a relationship | 757 (44%)  968 (56%) | 861 (62%)  526 (38%) | $\chi^{2}$(1)=101.2 | <0.001 | V=0.18 |
| Highest  Education  Level | Chi-squared | *n* (%) | 1747 | 1387 | Trade or Vocational  Secondary  University  Other | 672 (39%)  652 (37%)  348 (20%)  75 (4%) | 461 (33%)  278 (20%)  631 (46%)  17 (1%) | $\chi^{2}$(3)=276.9 | <0.001 | V=0.30 |
| Main route of Administration | Chi-squared | *n* (%) | 1450 | 1104 | Inhaled  Oral  Other | 1210 (83%)  216 (15%)  24 (2%) | 788 (71%)  293 (27%)  23 (2%) | $\chi^{2}$(2)=54.9 | <0.001 | V=0.15 |
| Preferred route of administration | Chi-squared | *n* (%) | 1080 | 929 | Inhaled  Oral  Other | 697 (64.5%)  354 (32.8%)  29 (2.7%) | 467 (50.0%)  414 (45.0%)  48 (5.0%) | $\chi^{2}$(2)=43.7 | <0.001 | V=0.15 |
| Had never used cannabis prior to using it for medical reasons | Chi-squared | *n* (%) | 1170 | 1109 | Had never used  Had used | 164 (14%)  1006 (86%) | 212 (19%)  897 (81%) | $\chi^{2}$(1)=10.4 | 0.001 | V=0.07 |
| Main condition being treated | Chi-squared | *n* (%) | 1629 | 1331 | Mental Health/Substance Use  Pain  Sleep  All others | 591 (36%)  582 (36%)  108 (7%)  348 (21%) | 437 (33%)  499 (37%)  123 (9%)  272 (20%) | $\chi^{2}$(3)=9.8 | 0.020 | V=0.06 |
| Main way medical cannabis is accessed | Chi-squared | *n* (%) | 1255 | 1044 | Recreational dealer  Friends or Family  Home grown  Accessed legally  All others | 578 (46%)  407 (32%)  142 (11%)  1 (0.1%)  127 (10%) | 482 (46%)  264 (25%)  121 (12%)  25 (2%)  152 (15%) | $\chi^{2}$(4)=46.3 | <0.001 | V=0.14 |

*V= Cramer’s V effect size estimate. Rules of thumb for Cramer’s V on chi-square tests: V < 0.1 = negligible, V ≥ 0.1 = small, V ≥ 0.3 = medium, V ≥ 0.5 large.*
